# A SAGES guide to navigating a changing landscape and continuing to promote surgical excellence and opportunity for all

**DOI:** 10.1007/s00464-025-12140-2

**Published:** 2025-08-28

**Authors:** Jenny M. Shao, Hope T. Jackson, Andrew Schlussel, Jacob Owen, Courtney Collins, Patricia Sylla, SAGES DLPD Committee, Jennifer Colvin

**Affiliations:** 1 Division of Minimally Invasive Surgery, Department of Surgery, University of Michigan2926A Taubman Center, 1500 E Medical Center Dr., Ann Arbor, MI 48109 USA; 2 HCA Florida Jacksonville Surgical Specialists, Jacksonville, FL USA; 3 Department of Surgery, East Carolina University, Greenville, NC USA; 4 Division of General Surgery, Department of Surgery, The Ohio State University, Columbus, OH USA; 5 Division of Colon and Rectal Surgery, Mount Sinai Hospital, New York, NY USA; 6 Society of Gastrointestinal and Endoscopic Surgeons, Diversity, Leadership, and Professional Development Committee, Los Angeles, CA USA; 7 Department of Surgery, University of Cinncinati, Cinncinati, OH USA

**Keywords:** Diversity, Equity, Inclusion, Policy change

## Abstract

**Background:**

Since January 2025, new executive orders and legislation have had wide impact on healthcare and the environment in which surgeons practice.

**Methods:**

The Society of American Gastrointestinal and Endoscopic Surgeons (SAGES) have reviewed the current executive orders, policies, and summarized its impact on our institutions, organizations, trainees, and patient care.

**Results:**

This article’s primary objective is to inform surgeons about how these policies and changes may impact them in their day-to-day practice in an effort to help them navigate these changes. The implications of these policies in healthcare settings, in Surgery Departments, and National organizations are still in flux as real world implementation is still being evaluated. It is important to understand each individual organization and institution, the federal and state laws, as the impact of these laws can vary dramatically across the geographic location and entity type. There are also many legal challenges to proposed executive orders and legislation making it difficult to understand when these policies will go into effect and if they are in fact legal.

**Conclusions:**

The long terms effects of the recent January 2025 legislation and executive orders are currently unknown. Increased knowledge, transparency, and understanding of these policies is crucial as organizations and institutions evaluate how to respond and navigate these changes.

As a national organization, the Society of American Gastrointestinal and Endoscopic Surgeons (SAGES) serves many roles, and one of those is a dedication and commitment to innovate, educate, and collaborate to advance patient care. We believe, both as an organization and as individuals, that this commitment should extend to all of our members, colleagues, and trainees regardless of their background or circumstance.

The current cultural and political landscape is changing rapidly, particularly around issues related to identity and inclusivity. As previously outlined in the SAGES Position Statement on diversity efforts, we believe that transparency and accountability are critical to maintaining systemic infrastructure that supports inclusivity within organizations [1]. This document serves as a commitment to support that transparency and to provide information on changes that can impact our professional environments. Along those lines this statement aims to do the following:

1. Outline current legislation and regulations around diversity, equity, and inclusion as of June 2025. Updates will continue to occur as this is a rapidly changing landscape.
2. Assess the potential impact of these changes on the practice of surgery, surgical education, training, as well as patient care.
3. Provide guidance on ways to navigate potential challenges in the times to come.

## What is the current landscape for diversity efforts?

Addressing issues around identity, inclusion, equity, and diversity has never been easy at any point in history and has come under increasing scrutiny in recent years. Productive conversations around these issues have proven challenging as the gap between political and ideological sensibilities continues to widen. Biases exist on both sides of the argument, and this is a social tension that has existed in this country since its inception and also extends to the wider global community. On one side, proponents of diversity, equity, and inclusion (DEI) will point out systemic inequalities that have led to differences in opportunities provided to certain underrepresented groups. The twentieth century was dominated by legislative efforts such as the Civil Rights Act of 1964, The Americans with Disabilities Act, The Equal Pay Act, and the Age Discrimination Act aimed to improve opportunities for all Americans regardless of their background. The importance of equity is also an established foundational principle in healthcare since it was identified as an essential quality domain by the Institute of Medicine in 2001 [2]. Regulatory bodies including the ACGME, JCHAO, and CMS have all implemented equity standards which require institutions to provide excellent care for all patients.

Alternatively, opponents of these efforts argue that diversity related initiatives are ineffective, cost prohibitive, and lack sufficient data to support their continued existence. Ironically, recent legal challenges to DEI initiatives have centered around the argument that DEI is, itself, discriminatory. In the 2023 Supreme Court decision Fair Admission vs. Harvard, the majority decision indicated that by using minority status to judge applicants, colleges were effectively using race to discriminate against the majority race (which has been identified as illegal in the past). Other legal challenges to DEI efforts have coalesced around similar arguments; that by distilling individuals down to their race/gender/ethnic identity, etc. and by using those criteria to distribute resources, institutions are inherently showing favoritism to one group over another.

It is in this precarious balance that we currently find ourselves. Adding to the confusion is the fact that a good deal of recent legislation does not define DEI and therefore it is difficult to know what exactly is being banned. How do we address the longstanding issues of racism, sexism, and religious discrimination (which are all still illegal) in the context of this new cultural and legal scrutiny? And, perhaps more importantly, how do we best continue to focus on providing the best possible clinical environment for our colleagues, patients, and trainees?

## What are the new executive orders regarding diversity, equity, and inclusion?

As of January 21, 2025, two new executive orders have been signed by the President specifically targeting diversity, equity, and inclusion initiatives. Taken together, these orders effectively ended funding for all DEI programs including scholarships, research, and training initiatives for federally funded efforts. The two orders are as follows:

- *Executive Order 14151: Ending Radical and Wasteful Government DEI Programs and Preferencing *[3]

This order mandates the termination of diversity, equity, inclusion, and accessibility (DEIA) initiatives within federal agencies. The order directs the Office of Management and Budget to eliminate all DEIA-related mandates, policies, programs, and activities across the federal government.

- *Executive Order 14173: Ending Illegal Discrimination and Restoring Merit-Based Opportunity *[4]

This order prohibits private organizations from conducting any DEIA employment programs for jobs created by federal contracts. The order nullifies multiple executive actions supporting DEIA, including affirmative action requirements for federal contractors and subcontractors (Executive order 11246 “Equal Employment Opportunity” signed in 1965 by President Lyndon B. Johnson), and directs agencies to remove all DEIA-related policies from hiring, training, and funding. In addition, all federal contracts and grants must certify that recipients do not operate DEIA programs which extends to scholarship funding.

Many other executive orders have also been enacted in this short time frame (Table 1). For the most updated and comprehensive list of recent executive actions please visit the White House communications page under “Presidential Actions” [5].

**Table 1:** Executive orders & policy changes impacting surgeons and the healthcare system

Executive order	Summary of policy changes	Status*
Executive Order 14,151: Ending Radical and Wasteful Government DEI Programs and Preferencing	Mandates the termination of diversity, equity, inclusion, and accessibility (DEIA) initiatives within federal agencies. The order directs the Office of Management and Budget to eliminate all DEIA-related mandates, policies, programs, and activities across the federal government	Challenged, Lawsuit and Court Hearings Pending
Executive Order 14,173: Ending Illegal Discrimination and Restoring Merit-Based Opportunity	Prohibits private organizations from conducting any DEIA employment programs for jobs created by federal contracts Nullifies multiple previous executive actions supporting DEIA, including affirmative action requirements for federal contractors and subcontractors	Challenged, Lawsuit and Court Hearings Pending
Executive Order 14,161: Protecting the United States from Foreign Terrorists and Other National Security and Public Safety Threats	Protect U.S. citizens from aliens who threaten national security, espouse hateful ideology, or otherwise exploit immigration laws for malevolent purposes Increased vigilance during visa-issuance process, increasing vetting and screening processes for immigrants who are already, or seeking admission to the U.S	None Currently
Executive Order 14,168: Defending Women from Gender Ideology Extremism and Restoring Biological Truth to the Federal Government	The U.S. recognizes two sexes male and female. These sexes are not changeable Eliminates access to gender-affirming medical care within the military healthcare facilities	Temporarily Blocked, Multiple lawsuits pending
Executive Order 14,187: Protecting Children from Chemical and Surgical Mutilation	The U.S. will not fund, sponsor, promote, assist, or support the so-called “transition” of a child (individual under 19 years) from one sex to another This includes discontinuation of hormone therapy, surgical procedures, related mental health support	Temporarily Blocked, Multiple lawsuits pending
National Institutes of Health (NIH) Policy Change NOT-OD-25–068	Seeks to cap indirect cost rate to 15% Indirect costs are part of the facilities and administration fees that help support research efforts	Temporarily Blocked, Multiple lawsuits pending

Many executive orders, challenges, and status of court hearings are ongoing and subject to change [25]

At this time, it is unclear if these executive orders will be upheld or enforced. As a result of these executive orders, many institutions (public and private) have dismantled DEI specific programming and initiatives. Others have kept existing programs but changed language and policy to be more inclusive of all and not limited to a specific underrepresented population. To date, there are a number of lawsuits filed against the executive orders and there will likely be additional court hearings about their constitutional validity and the power the executive branch of government has to implement them. The ultimate result may not be apparent for several months as these cases move through the judicial system.

## How can these new executive orders impact healthcare systems?

The recent executive orders eliminate federal support for DEIA programs, which will significantly impact state and university-affiliated medical centers, particularly those receiving federal funding (particularly Veterans Affairs (VA), grants, or contracts (Table 2). Although these orders primarily target federal agencies, they impose strict funding conditions that extend to state institutions and public medical centers. Institutions that rely on federal funding, such as NIH grants or Medicare/Medicaid reimbursements, may face new compliance requirements prohibiting DEI-based hiring, training, and programmatic initiatives if these new orders are upheld and implemented. If institutions do not comply, they risk losing federal funding, nonprofit status, or being subjected to investigations. Although every healthcare system will need to examine regulations at their state, local, and regional level, broadly speaking, the following practices may be revised (or eliminated).

- Requiring diversity statements on hire
- Prioritizing underrepresented candidates in the hiring process or distribution of leadership positions.
- Medical school admissions and scholarships must ensure compliance with the recent Supreme Court decision.
- Residency programs that provide support to underrepresented individuals may face restrictions or restructuring
- Educational content around cultural competence, implicit bias, or other “divisive ideologies” may face scrutiny depending on their structure.
- Elimination of diversity, equity, and inclusion from job titles, roles, speaking content, and programming
- State/University and Federal Medical centers will need to review policies to be race-neutral or face compliance investigations by the Attorney General and the Office of Management and Budget (OMB).

**Table 2:** Impact of executive orders on institution type

Institution type	Federal grants impact	DEI initiatives impact	DEI training impact	DEI hiring impact	Additional considerations
Department of Defense (DoD) & Veterans Affairs (VA)	Subject to strict compliance; risk of funding cuts if DEI policies are maintained	Eliminated; race-neutral policies mandated; no DEI-driven hiring or training allowed	Banned; all training must be race-neutral and focused on skills and competency	Strictly race-neutral; no affirmative action or DEI-based hiring considerations allowed	Military hospitals and VA facilities must strictly follow new mandates; failure to comply may lead to legal scrutiny
Academic hospitals with federal funding	Loss of federal grants for DEI-related research, training, and hiring initiatives	DEI programs must be dismantled or significantly altered to avoid funding issues	Elimination of mandatory DEI training programs for staff, residents, and students	Elimination of hiring quotas or preferences based on race, gender, or identity	Residency programs and medical schools tied to federal funding must revise DEI policies or risk funding loss
Non-federally funded academic hospitals	Not directly impacted; can still apply for private and non-federal grants	Can maintain DEI programs but may face external pressure to align with national trends	No direct ban but may adjust training based on national trends and best practices	Can maintain diversity hiring efforts but must ensure compliance with anti-discrimination laws	Institutions may still champion diversity through race-neutral strategies like socioeconomic-based recruitment
Community-based practices	Minimal to no federal grant impact unless linked to Medicare/Medicaid reimbursements	Can continue voluntary DEI initiatives without direct federal restrictions	Can continue offering DEI training without federal oversight	Not directly affected but may shift hiring strategies based on broader market trends	Patient demographics may drive DEI practices rather than federal mandates; institutions retain full discretion
Private surgical practices	No impact: private funding determines financial decisions	Completely independent of federal mandates; may maintain or eliminate DEI efforts as desired	DEI training is a business decision and remains unrestricted by federal policies	Independent of federal restrictions; can continue DEI hiring or remove it at their discretion	Market-driven approaches to DEI remain unaffected unless subject to external regulatory pressure
Nonprofit organizations	Risk of losing federal grants if DEI initiatives violate new mandates	DEI programs may need restructuring to avoid legal risks if receiving federal funds	If federally funded, DEI training must be neutral or risk legal challenges	Federal funding restrictions may require race-neutral hiring to avoid compliance issues	Nonprofits must balance their DEI goals with potential funding risks; compliance with state laws also matters
Private companies	Federal contractors (those who hold a federal contract) may lose funding if DEI initiatives violate new compliance rules	Federal contractors must eliminate DEI-driven hiring, training, and workforce balancing initiatives	DEI training must be restructured to comply with federal anti-discrimination policies	Federal contractors must ensure hiring is entirely merit-based and non-preferential	Private corporations must ensure compliance if they hold federal contracts but have flexibility otherwise
Surgical accreditation and professional societies (ex. ACGME, SAGES, ACS)	Federal grants for DEI-focused training and research at risk; accreditation-linked funding may be affected	May need to revise DEI programs to align with new federal standards, especially for leadership recruitment	May need to revise DEI training requirements to align with new accreditation standards	DEI-based leadership selection or faculty recruitment strategies may need to be adjusted	Accreditation requirements may shift to align with federal policies, influencing medical training programs

While these orders do not outright ban the concept of DEI programs, the strict limitations on funding, hiring, and compliance will make it challenging for institutions that rely on federal funding to sustain their initiatives. Additionally, the government has almost unlimited power over freedom of speech in government run institutions and properties which extends to its employees [6]. The reality is that even without an outright ban, medical centers will be pressured to alter or eliminate diversity programs, marking a significant shift in how targeted these programs can be in medical education and healthcare settings.

## How can these new executive orders impact nonprofit Surgical Societies such as SAGES?

For the time being, nonprofit organizations, particularly those that do not receive federal funding, grants, or contracts, can still operate independently, meaning that DEI initiatives can continue in the private sector. However, the Office of the Attorney General plans to target large nonprofits, foundations, and associations with assets over $500 million that continue to implement DEI-directed policies. Surgical societies and other nonprofits will need to consider that federal grants and partnerships may be reduced or eliminated for organizations that continue to support DEI-related programs. This may also be true for industry sponsorship depending on whether or not DEI continues to align with the corporation’s values. Scholarships, fellowships, and leadership programs aimed at underrepresented groups may need to shift toward race-neutral selection criteria, such as socioeconomic status or geographic diversity, rather than explicit underrepresented race or gender diversity-based eligibility. Regulations and interpretations of the current policies are still evolving but for the time being surgical societies and other nonprofits will need to determine for themselves whether DEI is still part of their core mission. Specifically for SAGES, many of the new DEI initiatives will not directly impact the organization. SAGES continues to stand by its commitment and previous position statement by continuing to emphasize the importance of diversity efforts and its positive impact on our mission, enhance opportunity for all members, and advocate on behalf of our patients.

## How do the executive orders affect the ACGME?

The Accreditation Council for Graduate Medical Education (ACGME) could face potential challenges surrounding DEIA efforts since many ACGME-accredited programs receive federal funding through Medicare Graduate Medical Education (GME) reimbursements, NIH grants, and VA hospital partnerships. These programs must ensure compliance with new federal regulations. As a result, diversity-focused residency recruitment efforts may need to realign toward race-neutral selection criteria, such as socioeconomic background, geographic diversity, or first-generation medical students, rather than explicitly prioritizing underrepresented groups. Additionally, ACGME’s DEI-related accreditation requirements for residency training and faculty development is currently undergoing evaluation. The ACGME board has suspended enforcement of certain diversity requirements pending internal board review set to occur in June 2025 [7]. These policies have already been mandated in military hospitals and could affect programs that are affiliated with the VA or receive federal funding.

## How will new immigration policies potentially impact the surgical workforce and how can we protect our international colleagues?

Although there is no singular executive order that specifically targets medical students, surgical residents, or practicing surgeons, multiple executive orders could indirectly affect clinicians who are not U.S. citizens practicing in the United States. The most critical executive order affecting this process is titled *“Protecting the United States from Foreign Terrorists and Other National Security and Public Safety Threats”*[8]*.* This executive order will increase scrutiny and extend the vetting process for visas, significantly delaying their approval. It is imperative for students, surgeons, and educators to have a general understanding of the visa process in order to support their colleagues. With this additional scrutiny on non-U.S. citizens, any visa holder or Green Card holders who are non-U.S. citizen legal permanent residents may also face additional security measures or questioning when entering the United States. These individuals face increased visa restrictions, may be at risk for their visa being revoked, a heightened vetting processes, and potential risks to completing their training, making institutional and legal support critical. For those who are applying for new visas, there are current suspension or partial suspensions for visa issuance to foreign nationals from certain countries [9]. Some steps that may help include the following:

- Become familiar with different types of visas and their requirements (Table 3).
- Provide easy and increased access to immigration attorneys ensuring trainees, students, and colleagues understand the requirements and remain compliant with evolving regulations.
- Establish institutional sponsorship programs to assist trainees in transitioning from J-1 to H-1B visas when applicable and should work with human resources and legal departments to ensure continued support despite changing federal policies.
- Look into other opportunities that allow individuals to remain in the US after completing training such as the Conrad 30 Waiver Program (Table 3).
- Evaluate the safety of international travel for non-US citizens while regulations are still unclear.

**Table 3:** Overview of common visa types and qualifications

Type of visa	Visa program	Who Qualifies?
J-1 Visa	Exchange visitor program	This is the most common visa for IMGs entering surgical residency programs: It is sponsored by the Educational Commission for Foreign Medical Graduates (ECFMG) and requires applicants to have passed USMLE Step 1 and Step 2 CK, obtained an ECFMG Certificate, and secured a residency position Requires a two-year home-country return requirement where the physician must return to their home country for two years after training unless they obtain a waiver; for example, Conrad 30 Waiver Program
J-1 Visa	Conrad 30 Waiver Program	The Conrad 30 Waiver Program is a federal initiative that allows IMGs on J-1 visas to remain in the U.S. after completing their residency or fellowship training without fulfilling the two-year home-country return requirement [26]: To qualify, the physician must have completed a U.S. surgical residency or fellowship. The service commitment requires three years of full-time employment at a designated healthcare facility Once approved, the physician can change from a J-1 visa to an H-1B visa, allowing them to remain and work legally in the U.S. After completing the three-year commitment, they become eligible to apply for a Green Card. Surgical trainees interested in this program should research state-specific requirements and secure a qualifying job offer early in their final year of training
F-1 Visa	Student Visa	Used primarily by international students attending U.S. medical schools: Allows full-time study but does not permit direct entry into residency training Students must transition to a J-1 or H-1B visa to pursue surgical residency
H-1B Visa	Specialty Occupation Visa	This visa allows medical graduates to work in a specialty field, including medicine, and is used by some residency programs as an alternative to the J-1 visa: This requires completion of all three USMLE steps, a state medical license, and program sponsorship There is no two-year home-country return requirement, making it a preferred option for those planning to stay in the U.S. after training Not all residency programs sponsor H-1B visas, and there is an annual cap on the number issued

Ensuring clear communication and emotional support is also essential, and institutions should create confidential support systems where trainees can seek guidance without fear of repercussions. Assigning faculty mentors familiar with visa policies can help provide both career and emotional support to affected trainees. By taking proactive steps, surgical educators can help minimize disruptions for visa-holding trainees and surgeons.

## What are the potential implications of new regulations on clinical practice for specific patient populations within general surgery?

The executive orders that may impact surgical clinical practice and our day-to-day care of patients center around new immigration and transgender care policies that may limit care to these specific patient populations. While these may not be patients we see on a regular basis, it is important to understand how these policies may affect access to care and potentially heighten existing healthcare disparities. There are many new policies in place impacting healthcare broadly including vaccinations and right to abortions, but they will not be discussed within the scope of this guide.

### Impact on transgender clinical care

Several new executive orders have been implemented that may impact Transgender clinical care including Executive Order 14,168 (limiting US recognition of gender to male and female only) [10] and 14187 (banning gender affirming care for transgender individuals under the age of 19) [11]. Taken together these orders represent a significant threat to the nonbinary and transgender population. The LGBTQIA + (Lesbian, Gay, Bisexual, Transgender, Queer or Questioning, Intersex**,** Asexual, and more) community already experiences high levels of discrimination throughout the healthcare system, especially transgender and nonbinary individuals. For this population, engaging in the healthcare system has always been a potentially traumatizing event, even before the recent cultural shifts [12]. Policies that limit care for this population will likely only discourage them further from engaging with the healthcare system.

Additionally, by limiting medical care for these individuals, we also will decrease physician comfort with addressing their unique needs. This impact could be felt not only when it comes to gender affirming care, but for all aspects of their health. Transgender medicine and its basic needs should be exposed to currently practicing physicians by continuing medical education to decrease health disparities among the lesbian, gay, bisexual, transgender, and queer community [13].

### Impact on undocumented immigrants

Similarly, immigration policies and executive orders targeting undocumented immigrants will also affect access to healthcare. Racial and ethnic minorities already have decreased access to care and are more likely to experience health inequities, including disparities in surgical care [14–16]. As a result, undocumented immigrants are more likely to rely on safety net hospitals and utilize emergency services [17]. Historically, Immigration and Customs Enforcement (ICE) avoided enforcement actions at sensitive locations like hospitals, acknowledging that patient care should take precedence over immigration enforcement [18]. However, under the current administration, immigration officials are being allowed increasing access to areas that were previously “off limits.” Furthermore, the increased presence of immigration authorities in some areas has made undocumented individuals (and their families who may in fact be citizens) reluctant to engage with state or federal institutions for fear of being detained. This means that even more than before, racial, and ethnic minorities may be at significant risk of falling through the cracks in our healthcare system. It is essential to remember that federal and state privacy laws prohibit the disclosure of patient information—including immigration status—to law enforcement. Immigration officers should not be allowed in private areas of the hospital without a warrant (Table 4). Healthcare workers should consult their hospital’s legal department when handling such situations [19].

**Table 4:** Health care providers & immigration enforcement

Possible actions	Implementation strategy
Establish a written policy identifying areas of the clinic/hospital as private and not open to the general public	Examples: patient care areas, exam rooms, offices, records areas
Designate a specific person responsible for handling contacts with law enforcement	Train all other staff to inform immigration or other law enforcement officials that only the designated persons are authorized to review a warrant or consent to entry to private areas
The designated person should state explicitly that they do not consent to entry into a private area without a warrant	Contact a lawyer to be present for the search
If presented with a warrant, the designated person should review it for validity	Without a valid warrant, they may NOT enter private areas To be valid it must: Be signed by a judge State the address of the specific premise to be searched
Provide posters, “Know Your Rights” cards, and educational materials	Remind patients they have a right to remain silent and NOT answer questions They should ask that a lawyer be present if they are questioned
Protect patient privacy	Patient information is protected under HIPAA ICE agents cannot access patient records without explicit authorization or judicial subpoena Do not disclose any patient information without consulting your hospital’s legal team
Know your rights as a worker	You have the right to remain silent and do not have to answer questions about your own immigration status Keep a record of the encounter, including names of the agents, the time and location, and any documents they present

## How will new National Institutes of Health (NIH) policies potentially impact research and NIH Funding?

In January, the current administration implemented a broad funding freeze across several federal agencies including the NIH, which was halted by two federal judges, and then quickly rescinded. In early February, the NIH announced a new policy that it would cut ‘indirect’ research or facilities and administrative costs to 15%, which effectively is a cut to support research [20]. These potential budget changes will reduce the capability of institutions and scientists to conduct research. At this time, a coalition of 22 states and research institutions have brought forth a lawsuit entitled “Commonwealth of Massachusetts v. National Institutes of Health” that blocks the NIH from instituting the change [21]. This court order and injunction has been extended and will be eventually heard in a court of law, preventing these policies from taking effect until the final judicial decision, which may take a year or longer to reach (Table 5).

**Table 5:** Proposed federal policy promoting diversity in education

Policy	Description	What Stage?
H.Res.3444—Strength in Diversity Act of 2023 [27]	Improve diversity and reduce or eliminate racial or socioeconomic isolation in publicly funded early childhood education programs, public elementary schools, or public secondary schools	Introduced: House of Representatives (5/2023)
H.R.1207—Diversity Advancements in Accelerated Programs Act [28]	Establish equity offices to improve diversity in accelerated learning programs (i.e., Advanced Placement), and carry out universal screening to determine eligibility for such programs	Introduced: House of Representatives (2/2023)
H.Res.608—Recognizing the importance of diversity, equity, and inclusion efforts in higher education [29]	Recognizes the importance of diversity, equity, and inclusion efforts at institutions of higher education	Introduced: House of Representatives (7/2023)
H.Res.1180—Recognizing the importance of diversity, equity, and inclusion efforts in medical education [30]	Expresses support for diversity, equity, and inclusion efforts at medical education institutions	Introduced: House of Representatives (4/2024)

It is likely that there will be additional proposals and bills introduced that will change funding priorities with a shift to prioritize Industries of the Future and National Security [22]. There may also be closer scrutiny to grants and new funding proposals that address areas of research not supported by the current administration (i.e., disparities, diversity) [23]. It is important to note that budgetary and policy changes happen frequently with changing policies and ideologies, but the full extent of what this means will be unclear for the time being.

## What can we do to navigate the coming changes?

Regardless of personal opinion, staying informed is critical to navigating the ever-changing and complex landscape as surgeons. Given the rise of misinformation and social polarization, it is essential to critically evaluate sources of information, where false narratives can be rapidly created, shared, and amplified through social media [24].​​ Although all of our perspectives and journeys will look different, we believe that we can agree on some common factors:

- Create a safe psychological space for people to be vulnerable and share in that vulnerability.
- Be open to conversations about current issues and listen without judgement.
- Avoid political and/or cultural discussions in public areas and particularly in the presence of individuals who may not feel free to express their opinion (particularly trainees and students).
- Reach out to colleagues, students, trainees, and patients who may be at risk of discrimination, trauma, or other adverse events during the coming months.
- Stay informed of regulations and understand the laws in your area and how they impact the people around you (for a comprehensive list of resources see Table 6).
- Avoid “knee jerk” reactions to legislation and executive orders whether proposed or enacted as many will undergo revision or clarification in the coming months.
- Engage with other national organizations to see how you can support their members. Some examples include:
  - Association of Women Surgeons (AWS)
  - National Hispanic Medical Association (NHMA)
  - Society of Black Academic Surgeons (SBAS)
  - Society of Asian Academic Surgeons
  - Latino Surgical Society (LSS)
  - Association of American Indian Physicians (AAIP)
  - Excelsior Surgical Society
  - Association of Out Surgeons and Allies (AOSA)
  - National Medical Association (NMA)

**Table 6:** Important websites and resources for federal and state information

Resource NAME	Website URL	What It Provides
Federal Register (Executive Orders)	https://www.federalregister.gov/presidential-documents/executive-orders	Official record of all presidential executive orders, including those affecting DEI and immigration
U.S. Equal Employment Opportunity Commission (EEOC)	https://www.eeoc.gov	Federal agency enforcing civil rights laws against workplace discrimination, including DEI policies
U.S. Citizenship and Immigration Services (USCIS)	https://www.uscis.gov	Official site providing immigration laws, visa regulations, and policy updates relevant to IMGs and medical professionals
National Conference of State Legislatures (NCSL)	https://www.ncsl.org	Comprehensive resource tracking state legislative actions, including DEI-related policies and state-specific laws
National Conference of State Legislatures (NCSL)	https://www.ncsl.org/research/civil-and-criminal-justice/affirmative-action-overview.aspx	Tracks state legislation on affirmative action and DEI policies, offering insights into state-specific DEI laws and initiatives
Government Alliance on Race and Equity (GARE)	https://www.racialequityalliance.org	Provides guides, tools, and resources for local and state governments to implement DEI strategies in hiring, contracting, and policymaking
Regional Government Services Authority (RGS)	https://www.rgs.ca.gov/diversity-equity-inclusion	Supports local and regional governments in implementing DEI action plans, decision-making guides, and policy frameworks
National League of Cities (NLC)	https://www.nlc.org/resource/cities-of-opportunity-dei-action-guide	Provides DEI best practices, case studies, and strategies for local governments and cities to promote inclusive policies
California DEI Initiatives	https://www.calhr.ca.gov/Pages/dei.aspx	Offers California state DEI initiatives, policies, training, and employment equity resources
New York State Office of Diversity and Inclusion	https://diversity.ny.gov/	Provides New York State DEI policies, diversity recruitment initiatives, and inclusion programs
Texas Office of Diversity, Equity, and Inclusion	https://www.hhs.texas.gov/about-hhs/leadership/office-diversity-equity-inclusion	Texas state DEI office focused on health and human services inclusion, workforce diversity, and training
Illinois Bureau of Diversity, Equity, and Inclusion	https://www.dhs.state.il.us/page.aspx?item=123667	Illinois state DEI initiatives, equity-focused workforce development, and policy implementation
Michigan Office of Diversity, Equity, and Inclusion	https://www.michigan.gov/odei	Michigan’s DEI policies and resources for state agencies and public institutions to promote diversity and inclusion
Washington—Municipal Research and Services Center (MRSC)—DEI Resources	https://mrsc.org/explore-topics/engagement/inclusion/diversity-equity-inclusion	Provides resources, tools, and sample documents related to DEI programs in local governments within Washington State
Florida—Equality Florida	https://equalityflorida.org/	A political advocacy group that advocates for civil rights and protections for LGBTQ residents in Florida. They offer resources and information on state-specific DEI initiatives
Virginia—Virginia Department of Human Resource Management—DEI Resources	https://www.dhrm.virginia.gov/diversity-opportunity-inclusion/dei-resources	Provides DEI resources specific to the state of Virginia, including policies, training materials, and initiatives
Ohio—Ohio State Bar Association—Diversity Initiatives	https://www.ohiobar.org/member-tools-benefits/practice-resources/diversity-initiatives/	Offers resources and information on DEI efforts within Ohio’s legal profession
New Mexico—New Mexico State University 4-H—DEI and Social Justice Resources	https://nm4h.nmsu.edu/resources/diversity.html	Provides DEI and social justice resources, including professional development and curriculum activities, specific to New Mexico
National Conference of State Legislatures (NCSL)	https://www.ncsl.org/research/civil-and-criminal-justice/affirmative-action-overview.aspx	Offers comprehensive insights into state policies and legislation related to affirmative action and DEI initiatives
National Governors Association (NGA)	https://www.nga.org/policy-positions/hsw-12/	Provides policy positions and resources on DEI efforts at the state level, reflecting the collective priorities of the nation’s governors
Council of State Governments (CSG)	https://www.csg.org/policy-areas/social-justice/	Focuses on social justice and DEI policies, offering resources and analysis pertinent to state governments

For the most accurate and up-to-date information, it’s advisable to consult your specific state’s government websites or DEI offices. Additionally, local universities, nonprofit organizations, and community groups often provide state-specific DEI resources and initiatives

The current landscape is changing rapidly, and we encourage everyone to stay informed, to not make assumptions, and to continue to come together to promote the values we all share. We continue to believe that surgical excellence, patient advocacy, and protecting every surgeon, trainee, and patient is paramount to ensuring a bright and inclusive future for our field.
